# ﻿Contribution to the knowledge of Teloganodidae (Ephemeroptera, Ephemerelloidea) of India

**DOI:** 10.3897/zookeys.1113.85448

**Published:** 2022-07-18

**Authors:** Alexander V. Martynov, T. Sivaruban, Dmitry M. Palatov, Pandiarajan Srinivasan, S. Barathy, Rajasekaran Isack, Michel Sartori

**Affiliations:** 1 National Museum of Natural History, National Academy of Sciences of Ukraine, Bohdan Khmelnytsky str., 15, 01030, Kyiv, Ukraine National Museum of Natural History Kyiv Ukraine; 2 PG & Research Department of Zoology, The American College, Madurai-625002, India The American College Madurai India; 3 Independent researcher, Lviv, Ukraine Unaffiliated Lviv Ukraine; 4 Department of Zoology, Fatima College, Madurai-625018, India Fatima College Madurai India; 5 Musée cantonal de zoologie, Palais de Rumine, Place de la Riponne 6, 1014 Lausanne, Switzerland Musée cantonal de zoologie Lausanne Switzerland; 6 Department of Ecology and Evolution, Biophore, University of Lausanne, 1015 Lausanne, Switzerland University of Lausanne Lausanne Switzerland

**Keywords:** COI, distribution, imago, larva, morphology, Pannota, Tamil Nadu, Uttarakhand

## Abstract

Two new species of *Dudgeodes* Sartori, 2008 and a new species of *Teloganodes* Eaton, 1882 are described from India; they are *Dudgeodesselvakumari* Martynov & Palatov, **sp. nov.** from Himalayan region (Uttarakhand), *Dudgeodesmolinerii* Sivaruban, Martynov, Srinivasan, Barathy & Isack, **sp. nov.**, and *Teloganodesbarathyae* Sivaruban, Martynov, Srinivasan & Isack, **sp. nov.** from the Tamil Nadu part of the Western Ghats. Thus, for now, the Teloganodidae fauna of India includes 11 species. *Dudgeodesselvakumari***sp. nov.** appears to be significantly extend northward the known distribution of *Dudgeodes*. Partial COI sequences were used as an initial clustering method to show the relationships of *D.selvakumari***sp. nov.** with other sequenced operational taxonomic units (OTU) of the genus.

## ﻿Introduction

The superfamily Ephemerelloidea is a relatively diverse group within Indian subcontinent. This article is the next contribution in a series of papers on the superfamily of the region. Ephemerelloidea has been actively studied during the last years, and, as a result, series of new species mainly of the family Ephemerellidae have been described from this territory (Selvakumar et al. 2014, 2018a, 2018b; Anbalagan et al. 2015; Martynov et al. 2019, 2021a, 2021b; Sivaruban et al. 2021; Srinivasan et al. 2021). Teloganodidae are less diverse than Ephemerellidae and more poorly studied in Indian subcontinent. Within India, the Teloganodidae were known until now by seven species in four genera: *Teloganodes* Eaton, 1882 (three species), *Dudgeodes* Sartori, 2008 (three species), *Derlethina* Sartori, 2008 (one species), *Indoganodes* Selvakumar, Sivaramakrishnan & Jacobus, 2014 (one species) (Selvakumar et al. 2014; Anbalagan et al. 2015; Srinivasan et al. 2021).

*Teloganodes* and *Dudgeodes* occur within Indomalayan realm only. *Teloganodes* consists of eight species distributed in the Indian subcontinent (Sartori et al. 2008; Selvakumar et al. 2014). Three of these species are known from India: *T. kodai* Sartori, 2008 and *T. dentatus* Navás, 1931 are endemic to the Western Ghats; *T. sartorii* Selvakumar, Sivaramakrishnan & Jacobus, 2014 is endemic to the Eastern Ghats (Selvakumar et al. 2018a). The genus *Dudgeodes* currently contains 16 species, and three of them are known from India: *D.bharathidasani* Anbalagan, 2015, *D.palnius* Selvakumar, Sivaramakrishnan & Jacobus, 2014, and *D.sartorii* Srinivasan, Sivaruban, Barathy & Isack, 2021 – all endemic to the Western Ghats (Sartori et al. 2008; Selvakumar et al. 2014, 2018a; Anbalagan et al. 2015; Martynov et al. 2016; Garces et al. 2020; Srinivasan et al. 2021).

Representatives of *Teloganodes* are well distinguished at larval stages from other genera of the family (Sartori et al. 2008). Nevertheless, the winged stages of teloganodids are poorly known, as they have been described for only two species: only the male imago is known for *T. dentatus* Navás, 1931 (the larval stage remains unknown), and the female imago and male subimago have been described for *T. tristis* (Hagen, 1858) (the larval stage remains unknown). Despite the large number of species of *Dudgeodes*, only four are known from the winged stages: *D.hutanis* Sartori, 2008 (female subimago, Indonesia), *D.ulmeri* Sartori, 2008 (male subimago, Indonesia), *D.lugens* (Navás, 1933) (female subimago, China), and *D.pescadori* Sartori, 2008 (male and female imago and subimago, Philippines) (Sartori et al. 2008). Thus, the winged stages of all Indian species have not yet been described.

In the present contribution we describe three new species from India: *Dudgeodesselvakumari* Martynov & Palatov, sp. nov. based on larval, imaginal, and egg stages; *Dudgeodesmolinerii* Sivaruban, Martynov, Srinivasan, Barathy & Isack, sp. nov. based on larval and egg stages, and *Teloganodesbarathyae* Sivaruban, Martynov, Srinivasan & Isack, sp. nov. based on the larval stage only.

## ﻿Materials and methods

Larvae were collected by hand picking and kick-net sampling in Uttarakhand Pradesh and Tamil Nadu, India. Winged stages were reared from larvae in Martynov-designed grow nets (Fig. 16A–C). All material is stored in 80–95% ethanol. Some specimens were mounted on slides with Canada balsam.

Specimens of *Dudgeodesselvakumari* sp. nov. and their body parts unmounted on slides were photographed using a Leica M205A microscope with a Leica Z16 APO apochromatic zoom system and Leica DFC450 camera. The photographs were processed with LAS Core v. 3.8. Body parts mounted on slides were photographed with a Ulab XY-B2T microscope with a Canon Power Shot A 630 camera. These photographs were processed with Adobe Photoshop CS5 and Helicon Focus v. 6. Larvae studied with a scanning electron microscope were dehydrated in ethanol and then critical-point dried. Scanning electron microscopy was done on a Vega3 Tescan SEM.

Larval morphological characters of *D.molinerii* sp. nov. and *Teloganodesbarathyae* sp. nov. were studied using a LABOMED Luzeo 6Z stereo zoom microscope with an AR 6 Pro camera. Specimens studied under SEM were dehydrated in ethanol and critical-point dried, then examined using a Zeiss EVO 18 SEM. The photographs were processed using Adobe Photoshop 7.0 when necessary.

Names of protuberances of thorax (excluding lateral anterior tubercles, LAs) are given according to Auychinda et al. (2020).

Type material on *D.selvakumari* sp. nov. is deposited in collection of first author in the National Museum of Natural History of the National Academy of Sciences of Ukraine, Kyiv, Ukraine (NMNH NASU; holotype and paratypes); collection of Dmitry Palatov (paratypes); Museum of Zoology, Lausanne, Switzerland (MZL; paratypes). The type specimens of *D.molinerii* sp. nov. and *T. barathyae* sp. nov. are deposited in the Zoological Survey of India, Southern Regional Centre (ZSI-SRC; Chennai, Tamil Nadu, India) and The American College Museum (AMC; Madurai, Tamil Nadu, India).

### ﻿Molecular study

Total genomic DNA was extracted from three specimens of *D.selvakumari* sp. nov. using the BioSprint 96 extraction robot (Qiagen Inc., Hilden, Germany) following the supplier’s instructions. We used the non-destructive protocol described by Vuataz et al. (2011), which enables post-extraction morphological study of specimens. We then amplified a 658-bp fragment at the 5′ end of the mitochondrial cytochrome c oxidase subunit I (COI) gene, corresponding to the standard animal barcode region, using the HCO2198 and LCO1490 primers (Folmer et al. 1994). Polymerase Chain Reaction (PCR) was conducted in a volume of 25 μl, consisting of 5 μl (unknown concentration) of template DNA, 1.3 μl (10 μM) of each primer, 0.2 μl (25 mM) of dNTP solution (Promega), 5 μl of 5× buffer (Promega) containing 7.5 mM of MgCl_2_, 2.5 μl (25 mM) of MgCl_2_, 1 U of Taq polymerase (Promega), and 9.7 μl of sterile ddH_2_O. Optimized PCR conditions included initial denaturation at 95 °C for 5 min, 40 cycles of denaturation at 95 °C for 30 s, annealing at 50 °C for 30 s, and extension at 72 °C for 40 s, with final extension at 72 °C for 7 min. Purification and automated sequencing was carried out in Microsynth (Balgach, Switzerland).

Alignment of analyzed sequences was made in BioEdit v. 7.0.5.3. Recently, both tree- and distance-based methods of species delimitation based on single-locus data have been used.

We calculated genetic distances within and between species and other taxa were calculated in MEGA v. 11 (Tamura et al. 2021). IQ-Tree and FigTree v. 1.4.4 were used for constructing phylogenetic trees from sequence data using a maximum-likelihood (ML) analysis. We used two models of molecular evolution: Tamura-Nei (TN93) (Tamura and Nei 1993) and Kimura 2-parameter (K2) (Kimura 1980) models with a gamma distribution (shape parameter = 0.19). This analysis involved eight nucleotide sequences. Codon positions included were 1^st^+2^nd^+3^rd^+Noncoding. All ambiguous positions were removed for each sequence pair (pairwise deletion option). There were 655 positions in the final dataset.

GenBank accession numbers for newly derived sequences are given in Table 1, with the nomenclature of gene sequences following Chakrabarty et al. (2013). Other used sequences of Teloganodidae were taken from Selvakumar et al. (2016), Garces et al. (2020), and GenBank (unpublished data). *Indoganodesjobini* Selvakumar, Sivaramakrishnan & Jacobus, 2014, *Teloganodessartorii* Selvakumar, Sivaramakrishnan & Jacobus, 2014, and *T. kodai* Sartori, 2008 were chosen as the outgroup (Selvakumar et al. 2016).

**Table 1.** Codes and origin of new sequences used in molecular study.

Species	Specimen catalogue number	Locality	GPS Coordinates	Date	GenBank ID	GenSeq nomenclature
*Dudgeodesselvakumari* sp. nov.	GBIFCH00970940	India, Uttarakhand, Mailani Range, vicinity of Garjiya village, unnamed river – left tributary of Kosi River	29.4732°N, 79.1640°E	1.v.2018	ON255658	genseq-2 COI
*Dudgeodesselvakumari* sp. nov.	GBIFCH00970941				ON255659	genseq-2 COI
*Dudgeodesselvakumari* sp. nov.	GBIFCH00970942				ON255660	genseq-2 COI

## ﻿Results and discussion

### ﻿Taxonomy

#### ﻿ Dudgeodes selvakumari

Taxon classificationAnimaliaEphemeropteraTeloganodidae

Martynov & Palatov sp. nov.

A2A8F9F4-D191-54EA-B0B4-BDF6032CE108

https://zoobank.org/20B9E573-1D83-40E0-9191-F26480F7A7CE

Figs 1 , 2 , 3 , 4 , 5 , 6 , 7 , 8

##### Material examined.

***Holotype***: imago ♂, with corresponding larval and subimaginal exuviae, India, Uttarakhand, Mailani Range, vicinity of Garjiya village, unnamed river – left tributary of Kosi River, 29.4732°N, 79.1640°E, 430 m a.s.l., 22.v.2018, A.V. Martynov & D.M. Palatov leg., Indi9Telsp/1 (NMNH NASU). ***Paratypes***: 27 larvae, 9 larvae exuviae, 6 imagos with subimaginal exuviae, including 5♂ and 1♀), ibid., 22.v.2018, A.V. Martynov & D.M. Palatov leg., Indi9Telsp/2–11 (NMNH NASU); 51 larvae, ibid., 1–2.v.2018, A.V. Martynov & D.M. Palatov leg. – NMNH NASU (25 larvae, Indi8Telsp/1–11), MZL (6 larvae), Palatov’s collection (20 larvae).

##### Etymology.

The new species is named in honour of Dr C. Selvakumar of India, who contributed significantly to the study of mayflies in India.

##### Description.

**Figure 1.**
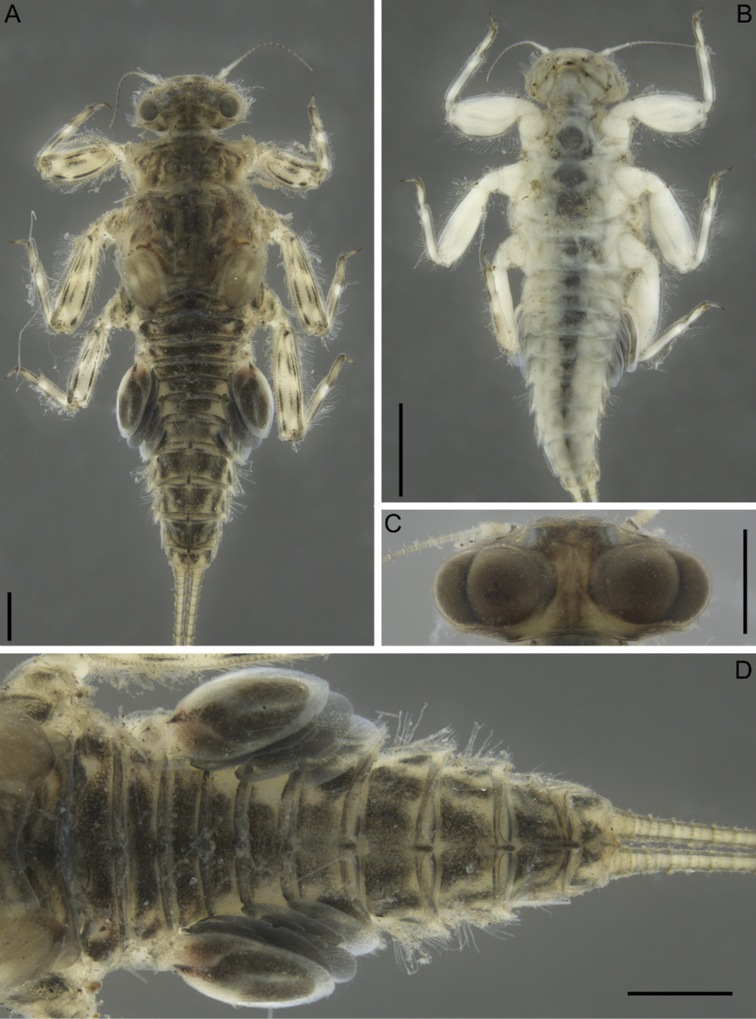
Larva of *Dudgeodesselvakumari* Martynov & Palatov, sp. nov., paratypes **A** total dorsal view **B** total ventral view **C** male head, dorsal view **D** abdomen, dorsal view. Scale bars: 0.5 mm (**A, C, D**); 1 mm (**B**).

***Mature larva*.** Body length 3.0–5.5 mm; cerci length 3.5–6.2mm. Dorsal surface of body yellowish with brown-black spots and strokes (Fig. 1A); ventral surface yellowish white, with indistinct median gray smudges on sternites (Fig. 1B).

***Head*** dirty yellow with indistinct brown smudges. Antennae also dirty yellow, distal segments of flagellum and distal part of scapus blackish. Dorsal part of male eyes brown (Fig. 1C). Occipital and suboccipital tubercles absent. Genae moderately developed. Antennae length 1.15 times head width, flagellum with about 15 or more segments. Lateral margin of head fringed with a row of long, stout setae, forked near base and with pointed apices, a row extending from posterior margin of eyes to labrum; stout setae on posterior margin of eyes distinctly shorter (Fig. 3B). Head covered with scattered, short, hair-like setae and short, stout setae with slightly divided margins.

**Figure 2.**
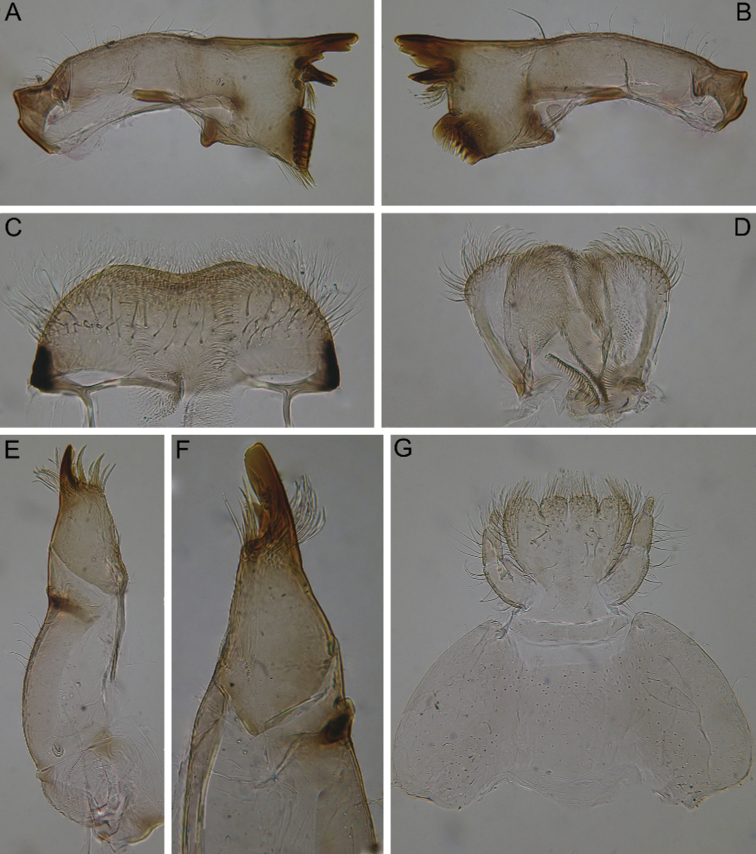
Larva of *Dudgeodesselvakumari* Martynov & Palatov, sp. nov., paratypes **A, B** mandibles **C** labrum **D** hypopharynx **E** maxilla **F** apical part of maxilla **G** labium.

***Mouthparts*.** Labrum wide and compact, width/length ratio 2.64–2.65, with smooth medial concavity on anterior margin (Fig. 2C). Dorsal surface covered with transversal band of long, stout, hair-like setae. Anterior area and margin densely covered with variously sized feathered setae.

Mandibles slender with few small setae along outer margin and one stout, hair-like seta in middle of margin. Right mandible (Fig. 2A): outer incisor composed of three teeth, one of them located remotely from others; inner incisor with two teeth; prostheca reduced, consisting of a bunch of thin setae; row of 6–8 long, stout, hair-like setae below mola and some short setae above mola. Left mandible (Fig. 2B): outer incisor with three teeth; inner incisor with two subequal teeth inserted transversely; prostheca small with a group of small setae; no setae below mola; base of mola with 2–3 small, apically pointed, stout setae.

Maxilla (Fig. 2E, F) slender, with well-developed canine, two dentisetae, and four long stout setae on inner apical part; crown with bunch of long setae; inner margin of lacinia base with 1+4 feathered, long, stout setae; maxillary palp reduced to a protuberance with single hair-like seta.

Hypopharynx with long, feathered setae on the rounded apexes of superlinguae, and very short setae on lingua (Fig. 2D).

Labial palp three-segmented, slightly constricted towards apex; articulation between segments clearly visible; segment III elongate and rounded apically, length/width at base ratio 1.9–2.2 (Fig. 2G). Outer margins of segments I and II covered with sparse, long, stout, hair-like setae; segment III with several fine setae only. Submentum well developed laterally. Glossae and paraglossae short and broad, rounded apically, their apexes densely covered with variously sized, feathered, stout setae; outer margins of paraglossae covered with long, feathered, stout setae.

**Figure 3.**
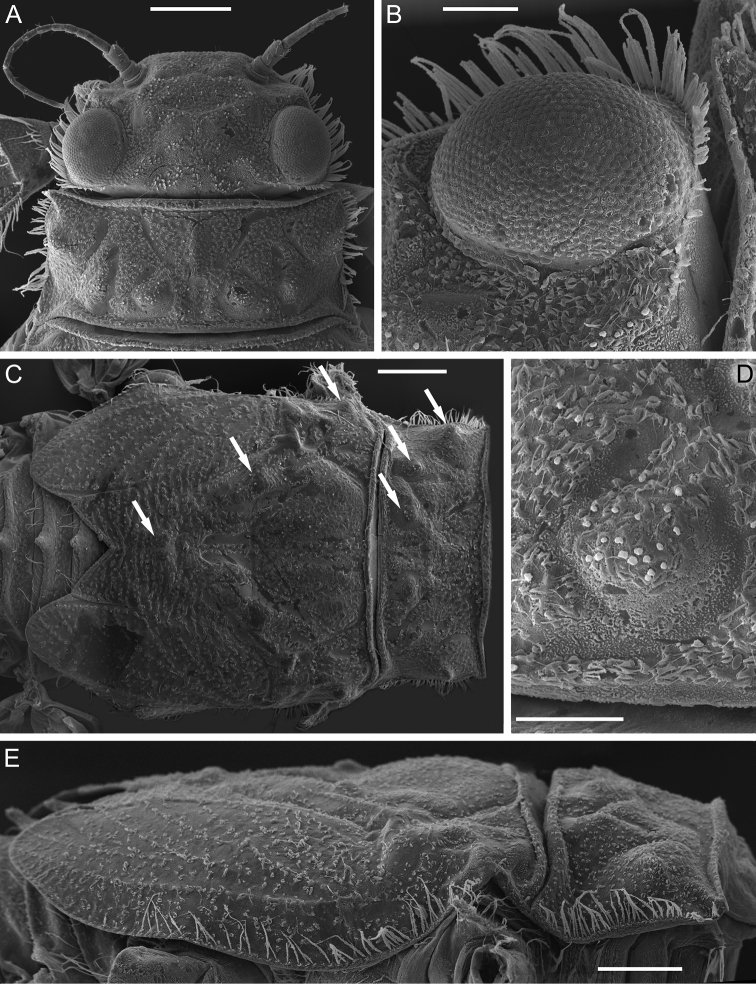
Larva of *Dudgeodesselvakumari* Martynov & Palatov, sp. nov., paratypes **A** head and pronotum, dorsal view **B** row of setae at outer margin of head **C** thorax, dorsal view **D** sub-median tubercle (SM) of pronotum **E** thorax, lateral view. Abbreviations: white arrows show tubercles of pronotum and mesonotum. Scale bars: 0.3 mm (**A, C, E**); 0.1 mm (**B, D**).

***Thorax*.** Pronotum with three pairs of tubercles: SMs, SLs, and Ls; with a few short globular stout setae; M tubercle absent (Fig. 3C–E). Lateral margins of pronotum and mesonotum with a row of long, stout setae, some of them forked (Fig. 3E). Surface of mesonotum with an MP, pair of SMMs and pair of LAs (lateral anterior tubercles) (Fig. 3C, E); these tubercles also bear a few short, stout setae with slightly divided margins.

**Figure 4.**
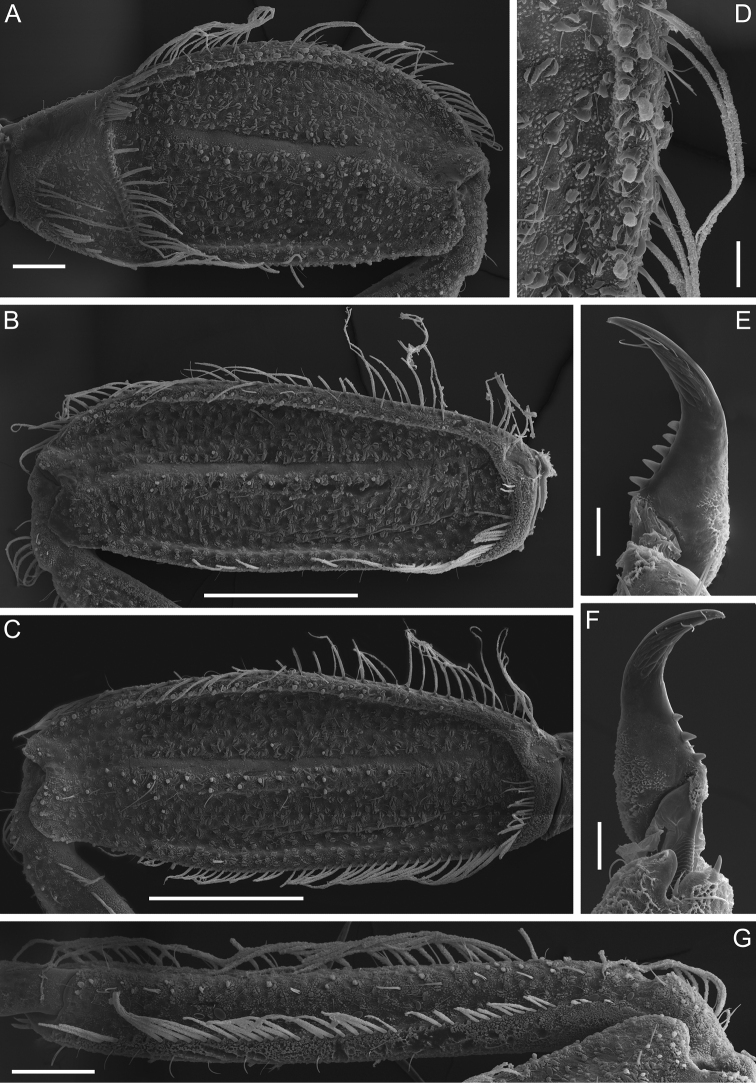
Larva of *Dudgeodesselvakumari* Martynov & Palatov, sp. nov., paratypes **A** fore femur **B** middle femur **C** hind femur **D** outer margin of fore femur **E, F** tarsal claws **G** middle tibia. Scale bars: 0.1 mm (**A, G**); 0.3 mm (**B, C**); 0.03 mm (**D–F**).

Forefemur moderately slender, ca 2.1 times longer than wide; outer margin covered with regular row of long, stout, hair-like setae, and few thin, hair-like setae (some setae in bunches); submarginal row of setae distinct, composed of stout setae (elongate and short) with slightly divergent margins (Fig. 4D); inner margin with row of long, stout, hair-like setae. Transverse row on dorsal surface consists of about 20 long, apically pointed, stout setae (Fig. 4A). Irregular row of short, stout setae with slightly divergent margins located parallel to longitudinal indistinct ridge (Fig. 4A–C). Dorsal surface of fore tibia with few scattered, short, stout setae of same kind, solitary hair-like setae, and hair-like setae in bunches (consisting of 2–4 setae), oblique regular row of long, stout, hair-like setae. Outer and inner margins of tibia with relatively short hair-like setae; inner margin with several elongate, pointed, stout setae.

Middle and hind femora (Fig. 4B, C), in contrast to fore femur, more slender, ca 2.6–2.7 times longer than wide, with denser submarginal row of stout setae, inner margin with regular row of long, stout, hair-like setae. Outer margins of middle and hind femora and tibiae with a regular row of long, stout, hair-like setae (Fig. 4D, G). Setation of dorsal surface of middle and hind tibiae most similar to those of fore leg, but oblique regular row of long, stout setae longer, and reaching distal end of tibia (Fig. 4G).

Tarsal claws moderately hooked, lacking subapical denticles, with 4–6 medial denticles and several (3–5) subapical setae (Fig. 4E, F).

**Figure 5.**
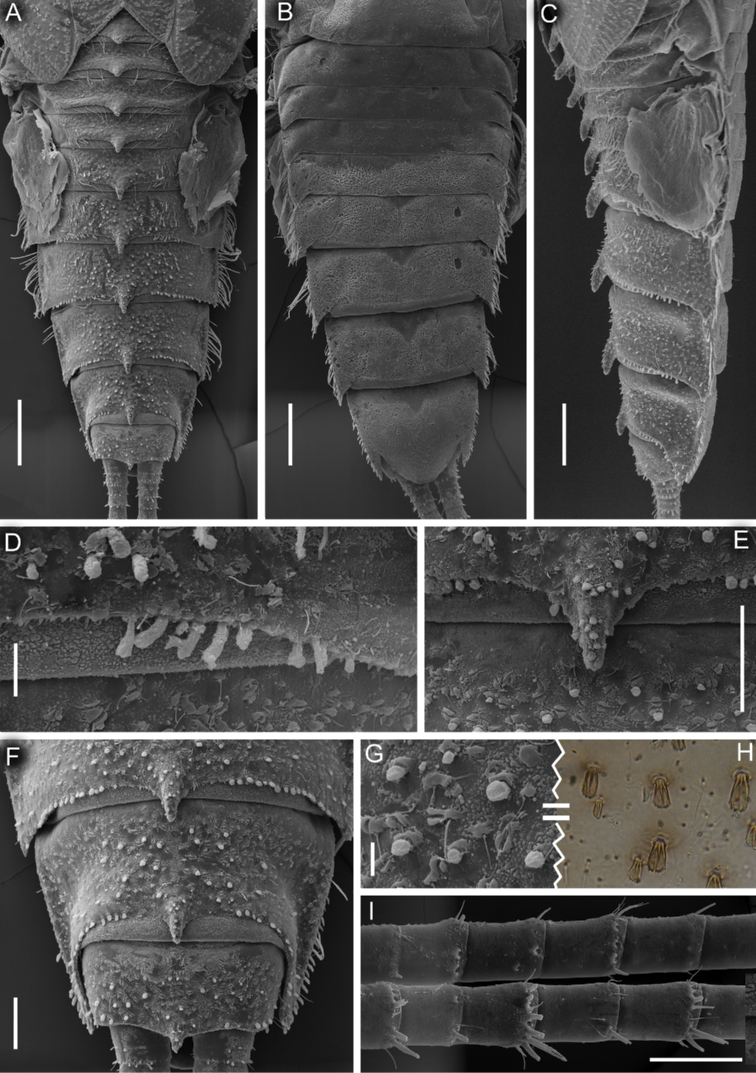
Larva of *Dudgeodesselvakumari* Martynov & Palatov, sp. nov., paratypes **A–C** abdomen, dorsal **A** ventral **B** and lateral **C** views **D** sublateral area of posterior margin of tergum VI **E** median tubercle of tergum VII **F** terga VIII–X, dorsal view **G, H** dorsal surface of tergum VIII, SEM microscopy **G** and light microscopy (**H**) **I** caudal filaments. Scale bars: 0.3 mm (**A–C**); 0.03 mm (**D, G**); 0.1 mm (**E, F, I**).

***Abdomen*.** All terga with moderately developed, narrowed (especially on terga V–X) median tubercles (Fig. 5A, F); the largest on terga V–VIII; tubercle of tergum X narrow and pointed. Median tubercles of terga I–IX distinctly elongate in lateral view (Fig. 5C). Median tubercles covered with short stout setae with divergent margins (Fig. 5E). Posterolateral projections moderately developed on segments VI–IX, and slightly marked on segments II–V (Fig. 5B).

Posterior margin of terga I–V with row of long, stout, hair-like setae; posterior margin of terga VI–IX with row of elongate (on tergum VI) and short (all other terga) stout setae with rounded apices (Fig. 5D); posterior margin of tergum X without stout setae (Fig. 5F). Mainly median area of terga with scattered short stout setae with divergent margins; most numerous on segments VI–X (Fig. 5G, H). Lateral areas of dorsal surfaces of terga III–VI with thin, hair-like setae and long, stout setae with serrated margins and apices. Narrow teeth present on posterior margin of submedian area of terga III–IV; the same teeth present on median and lateral areas of posterior margin of terga V–VI (they are not numerous at lateral areas) (Fig. 5D) and across entire posterior margin of terga VII–X (on tergum X not numerous); this kind of teeth absent on posterior margin of terga I–II. Sterna surface with a few scattered hair-like setae (Fig. 5B).

**Figure 6.**
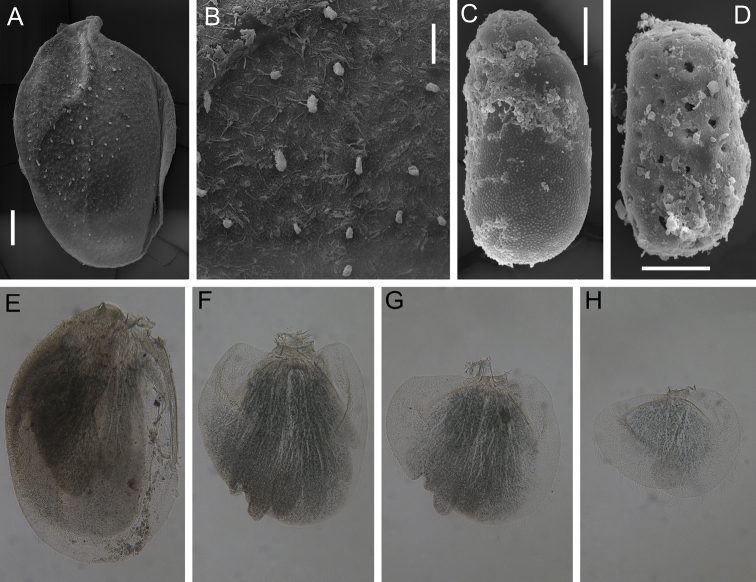
Larva of *Dudgeodesselvakumari* Martynov & Palatov, sp. nov., paratypes **A, E** gill II **B** dorsal surface of gill II **C, D** egg **F** gill III **G** gill IV **H** gill V. Scale bars: 0.1 mm (**A**); 0.03 mm (**B–D**).

Gills on segments II–V (Fig. 6A, E–H); gill II with dorsal lamella operculate, oval and with entire margin, mainly basal half covered with scattered short stout setae (Fig. 6B); gills III–V with dorsal lamella incised medially.

Cerci length subequal to the body length, posterior margins of central segments with hair-like and forked stout setae; length of the stout setae less than length of corresponding segment (Fig. 5I). Paracercus absent.

***Subimagos*.** Wings wholly grey, semitransparent in subimagos of both sexes.

**Figure 7.**
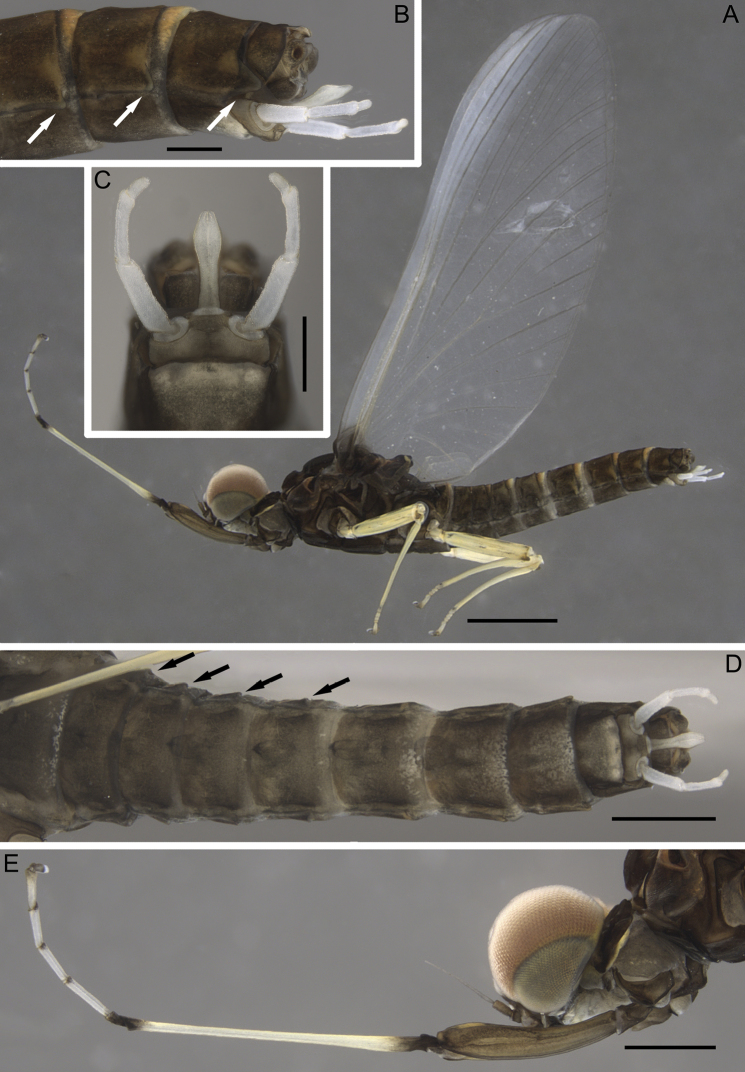
Imago male of *Dudgeodesselvakumari* Martynov & Palatov, sp. nov., holotype **A** total lateral view **B** abdominal segments VII–X, lateral view **C** genitalia, ventral view **D** abdomen, ventral view **E** head and fore leg, lateral view. Abbreviations: white arrows show postero-lateral projections of segments; black arrows show remnants of gill sockets. Scale bars: 1 mm (**A**); 0.2 mm (**B, C**); 0.5 mm (**D, E**).

***Male imago*.** Body length: 5.6–6.5 mm; forewing length: 5.6–5.9 mm; cerci length: 11.0–13.3. General coloration brown; thorax dark brown. Turbinate eyes brick-colored (Fig. 7A, E).

Fore leg (Fig. 7E): coxa, trochanter, and femur brown; tibia whitish, its basal and distal ends distinctly marked with black; tarsus segment I blackish; tarsi segments II–V whitish, with slightly blackish distal ends; tarsal claws blackish. Middle and hind legs (Fig. 7A): coxa brown, all other parts yellowish; femora with narrow, intermittent longitudinal black line along outer margin; dorsal surface with several longitudinal indistinct smudges; lower protuberance of knee brown; distal end of tibia blackish. Both claws on fore leg blunt; inner claw at middle and hind legs hooked and pointed and outer claw blunt.

**Figure 8.**
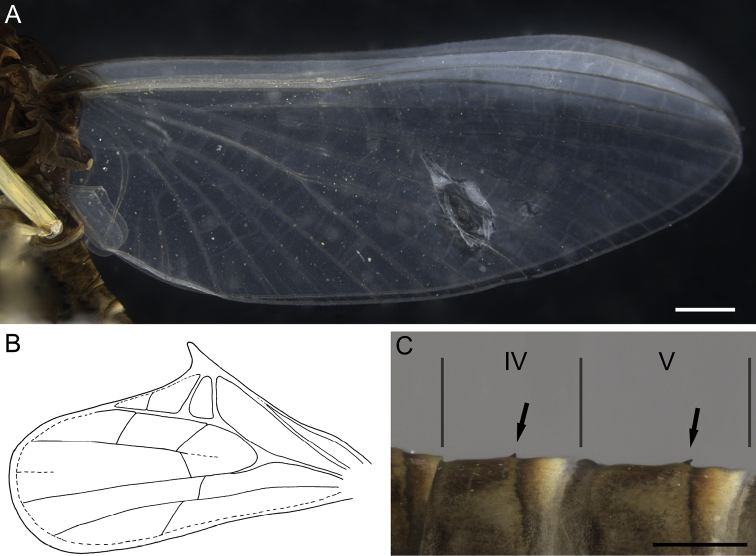
Imago male of *Dudgeodesselvakumari* Martynov & Palatov, sp. nov., holotype **A** wings **B** hind wing **C** abdominal segments IV and V, lateral view. Scale bars: 0.5 mm (**A**); 0.2 mm (**C**).

Main area of fore wing transparent (Figs 7A, 8A); only basal area with a black marking, costal and subcostal fields translucent, milky. Pterostigmatic area with 8–10 cross-veins, several of them divided. Hind wing elongate, with large costal process. Three or four cross-veins between Sc and RA; two cross-veins between RA and IRA (Fig. 8B).

Abdominal terga IV–VIII with small pointed median tubercles, in some specimens these tubercles distances on terga IV–V only (Fig. 8C). Abdominal segments VI–IX with distinct rounded apically postero-lateral projections, largest on segments VIII and IX (Fig. 7B). Segments II–V with remnants of gill sockets (Fig. 7D). *Genitalia*: whitish; styliger plate straight to concave; forceps 3-segmented; segments I and II approximately same length; segment I subcylindrical; segment II slightly expanded at apex; segment III rounded apically, elongate, 1.8–1.9 times as long as wide. Penis lobes with rounded apices; lobes expanded closer to apices, maximum width on ca 0.7 of their length; fused for entire length except the apex; on ventral side a groove ends at the middle of the penis (Fig. 7B, C).

***Female imago*.** Body length: 6.0 mm; forewing length: 5.9 mm; cerci length: 12.8 mm. General coloration brown. Legs coloration as in male imago. Turbinate eyes brown.

On fore leg outer tarsal claw hooked and pointed and inner claw blunt. On middle and hind legs outer tarsal claw blunt and inner hooked and pointed. Wings venation as in male imagos, but longitudinal veins browner. Only abdominal segments VII–IX with distinct rounded apically postero-lateral projections, largest on segments VIII and IX. Abdominal terga IV–VIII with small pointed median tubercles. Tergum X with longitudinal distinctly divergent median concavity that reach posterior margin. Segments II–V with remnants of gill sockets. Subgenital plate not elongate, with wide and shallow concavity. Subanal plate rounded.

***Egg*** (dissected from mature larva). Shape (Fig. 6C) ovoid, with one polar cap; chorion lacking attachment structures; without geometrical marcorelief, only microgranules present. Another kind of observed eggs (Fig. 6D) we considered as unformed yet; lacking polar cap, microgranules on chorion indistinct; whole surface covered with numerous depressions and holes.

##### Distribution.

Himalaya (Uttarakhand, India). All Indian representatives of *Dudgeodes*, excluding *D.selvakumari* sp. nov., are known from the Western Ghats only. *Dudgeodesselvakumari* sp. nov., which is distributed in the lower down part of Great Himalayan mountain range, is the most northern representative of the genus and family within India. This new species and *D.lugens* (Navás, 1933), which is known by single female subimago from Zhou Shan Island, in Zhejiang province, China (Sartori et al. 2008), are the most northern representatives of the family anywhere.

##### Habitats.

Larvae of this species were collected in a mid-sized river (6–10 m wide) in a shallow woodland valley at an altitude of about 400 m a.s.l. in the southern foothills of the Great Himalaya Range (Nainital District, Uttarakhand state, India). The river was relatively warm (24–26 °C), had a current of moderate velocity (ca 0.3–0.7 m/s), and was with a mainly stony or rocky substrate. The river is located in the recreational zone of the Jim Corbett National park with a weak anthropogenic load. Larvae were collected from the riparian zone from stones or vegetation at local current velocity 0.05–0.2 m/s (Fig. 16A–D), along with different *Baetis* sp. (Baetidae), Heptageniidae, *Choroterpes* sp. (Leptophlebiidae), *Caenis* sp. (Caenidae), *Asiagomphus* sp. (Gomphidae), *Macromyia* sp. (Macromiidae), *Protohermes* sp. (Corydalidae), *Agapetus* sp. (Glossosomatidae), *Chimarra* sp. (Philopotamidae), *Marilia* sp. (Odontoceridae), and *Macrobrachium* sp. (Palaemonidae).

##### Diagnosis.

The new species can be distinguished from other representatives of the genus by the following combination of characters. *Larva*: (i) dorsal part of male eyes brown; (ii) antennae length 1.15 times head width, flagellum with about 15 or more segments; (iii) labrum with transversal band of long, stout, hair-like setae; (iv) prothorax with three pairs of tubercles: SMs, SLs, and Ls; mesothorax with an MP, pair of SMMs and pair of LAs; (v) forefemur without transversal row of stout setae; (vi) outer margin of forefemur covered with a regular row of long, stout, hair-like setae, a few bunches, and single, thin, hair-like setae; (vii) submarginal row of setae of forefemur distinct, consisting of elongate and short, stout setae with slightly divided margins; (viii) tarsal claw of all legs with 4–6 medial denticles and without subapical denticles; (ix) terga I–X with moderately developed, narrowed (especially on terga V–X) median tubercles; the largest tubercles on terga V–VIII; tubercle of tergum X narrow and pointed; in lateral view, median tubercles of terga I–IX distinctly elongate; (x) posterolateral projections moderately developed on segments VI–IX, slightly marked on segments II–V. *Imago male*: (i) fore wing with numerous cross-veins; (ii) hind wing with 3–4 cross-veins between Sc and RA, and two cross-veins between RA and IRA; (iii) penis lobes maximum width on about 0.7 of their length; (iv) abdominal terga IV–VIII with small pointed median tubercles; in some specimens these tubercles distinct on terga IV–V only; (v) abdominal segments VI–IX with rounded apically postero-lateral projections. *Egg*: (i) without spines on pole opposite to polar cap; (ii) surface covered with microgranules.

The larva of *D.selvakumari* Martynov & Palatov, sp. nov. is easily distinguished from other Indian *Dudgeodes* species by: (i) absence of tubercles on head; (ii) number of tubercles on pro- and mesonotum; (iii) forefemur setation; (iv) shape of fore femur; (v) absence of subapical denticles on tarsal claws; (vi) shape of gill II; (vii) shape of median tubercles of abdominal terga.

#### ﻿ Dudgeodes molinerii

Taxon classificationAnimaliaEphemeropteraTeloganodidae

Sivaruban, Martynov, Srinivasan, Barathy & Isack sp. nov.

CC90A06F-686C-5391-8E0A-26CFF2F627DB

https://zoobank.org/060B341B-E366-4FC0-96B4-C81BE326FAF4

Figs 9 , 10 , 11

##### Material examined.

***Holotype***: mature ♀ larva, India, Tamil Nadu, Theni district, Kurangani hills, Kottakudi River, 10.0809°N, 77.2552°E, 632 m a.s.l., 28.x.2020, Pandiarajan Srinivasan & Isack Rajasekaran leg., ZSI–SRC/I/E/654. ***Paratypes***: 5 larvae, ibid., 28.x.2020, Pandiarajan Srinivasan & Isack Rajasekaran leg., ZSI–SRC/I/E/655 (1 larva), AMC ZN 237 (4 larvae).

##### Etymology.

The new species is named in honour of Dr Carlos Molineri of Argentina, who contributed significantly to the study of mayflies.

##### Description.

**Figure 9.**
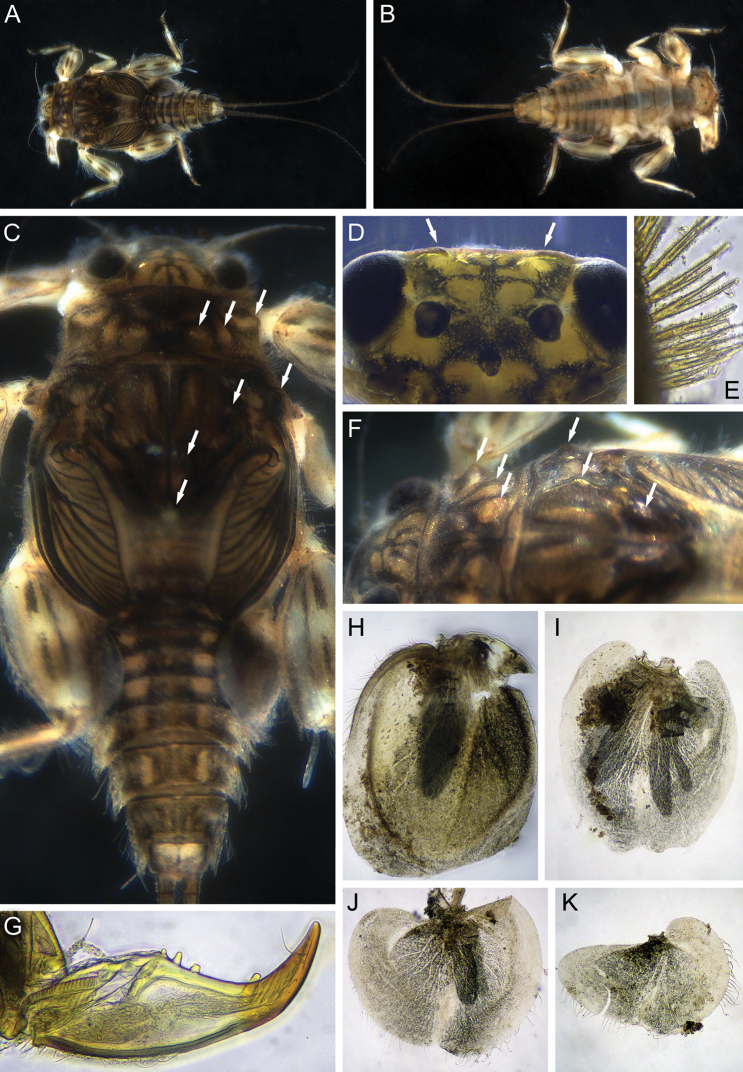
Larva of *Dudgeodesmolinerii* Sivaruban, Martynov, Srinivasan, Barathy, Isack, sp. nov., paratypes **A** total dorsal view **B** total ventral view **C** head, thorax and abdomen, dorsal view **D** head, dorsal view **E** row of setae at outer margin of head **F** head and thorax, dorso-lateral view **G** tarsal claw **H** gill II **I** gill III **J** gill IV **K** gill V. Abbreviations: white arrows show tubercles.

***Mature larva*.** Body length 4.7–4.9 mm; cerci length subequal to body length. General coloration of the dorsal side of head dirty yellow, with dark brown to blackish maculations; thorax and abdomen dark brown to blackish, with dirty yellow maculations (Fig. 9A–D). Ventral side of body yellowish to light brown. Dorsal surface of femora with two blackish longitudinal stripes one medial along ridge and one along outer margin; also three (proximal, medial, and distal) black spots with indistinct borders along medial ridge; proximal and medial spots divided in two parts by brownish bands (Fig. 11A–C). Dorsal part of male eyes dark brown to black (Fig. 9D). Abdominal terga I–VIII with pair of submedian yellowish spots. Anterior part of tergum X yellowish (Fig. 9C).

***Head*** with pair of occipital tubercles (Fig. 9D). Genae moderately developed. Lateral margin of head capsule from eye to labrum insertion with row of long, forked near the base, stout setae with pointed apices (Fig. 9E). Antennae length 1.25 times head width, flagellum with 11 segments. Head covered with scattered short hair-like setae and short stout setae with slightly divergent margins.

**Figure 10.**
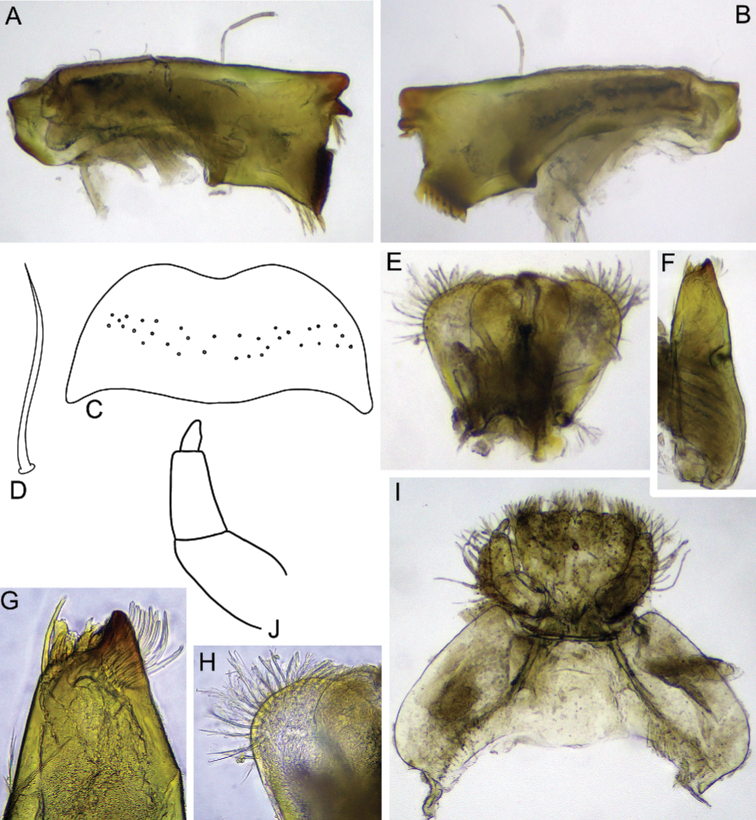
Larva of *Dudgeodesmolinerii* Sivaruban, Martynov, Srinivasan, Barathy & Isack, sp. nov., paratypes **A, B** mandibles **C** labrum **D** stout setae of transversal row of labrum **E** hypopharynx **F** maxilla **G** apical part of maxilla **H** superlingua **I** labium **J** labial palp.

***Mouthparts*.** Labrum compact, width/length ratio 2.51–2.53; with smooth anterior emargination; dorsal surface with transversal row of scattered, stout, hair-like setae (Fig. 10C, D); anterior area and margin of labrum densely covered with differently sized feathered setae. Mandibles slender with long, stout, hair-like seta in the middle of the outer margin (Fig. 10A, B). Number of teeth of both mandibular outer incisors cannot be determined precisely due to their wear in type specimens. Right mandible inner incisor with two teeth; prostheca reduced, with the appearance of a cluster of thin setae; small row of five long, stout, hair-like setae below mola and some short setae above mola. Left mandible inner incisor with two teeth inserted transversely, one smaller and rounded and other one larger and rounded; prostheca small; no setae below and above mola. Maxilla (Fig. 10F, G) slender, shape of canine impossible to determine (completely worn); two indented dentisetae and three long setae on inner apical part and cluster of long, simple setae at crown; inner margin at the base of lacinia, with two feathered, long setae; maxillary palp highly reduced to protuberance. Hypopharynx (Fig. 10E, H) with long, feathered setae on the rounded apexes of superlinguae and very short setae on lingua. Labial palp (Fig. 10I, J) three-segmented, slightly constricted towards apex; articulation between segments clearly visible; segment III elongate and rounded apically, length/width ratio at base 2.0–2.1. Surface and margins of segments I and II covered with scattered long, stout, hair-like setae; segment III bare. Submentum well developed laterally. Glossae and paraglossae short and broad, rounded apically, their apexes densely covered with differently sized, feathered, stout setae; outer margins of paraglossae covered with long feathered setae.

***Thorax*.** Pronotum with three pairs of tubercles: SMs, SLs, and Ls; tubercles with a few short, rounded setae. Mesonotum with three pairs of tubercles: two pairs of SMMs, a pair of LAs, and unpaired MP (Fig. 9C, F).

**Figure 11.**
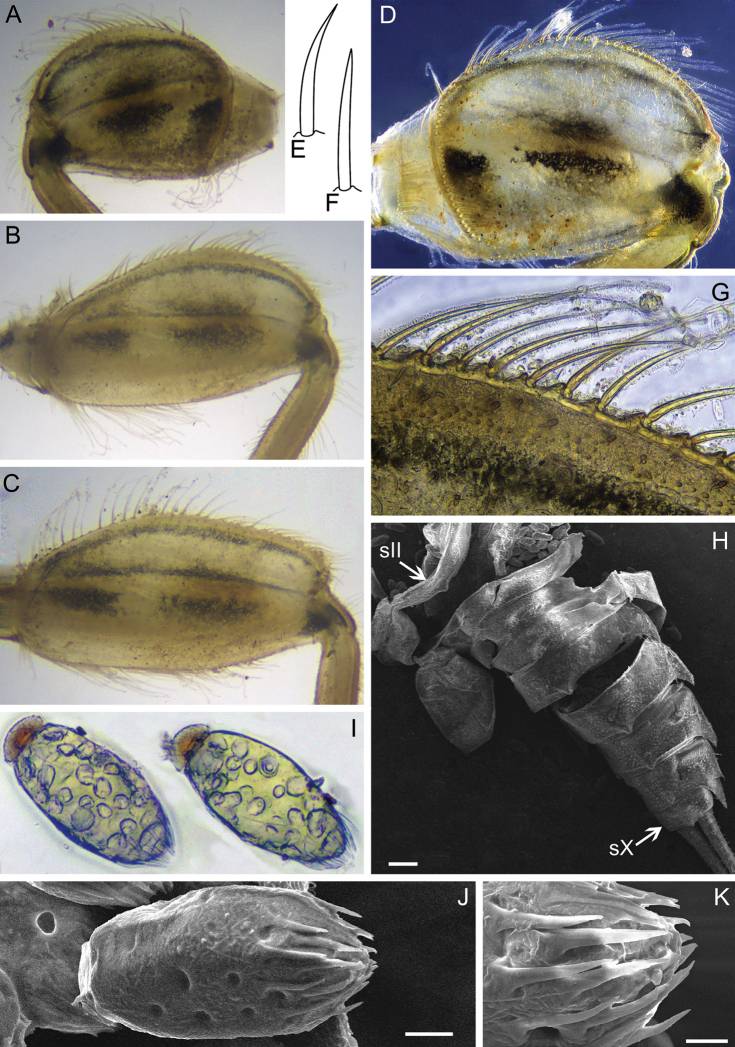
Larva of *Dudgeodesmolinerii* Sivaruban, Martynov, Srinivasan, Barathy & Isack, sp. nov., paratypes **A, D** fore femur **B** middle femur **C** hind femur **E, F** stout setae of transversal row on forefemur **G** outer margin of fore femur **H** abdomen **I** eggs, light microscopy **J** egg, SEM microscopy **K** cluster of spines on pole of egg. Abbreviations: sII – abdominal segment II, sX – abdominal segment X. Scale bars: 0.2 mm (**H**); 0.02 mm (**J**); 0.01 mm (**K**).

Forefemur broad, ca 1.3 times longer than wide (Fig. 11A, D); outer margin covered with a row of long, stout, hair-like setae (Fig. 11G); submarginal row of setae composed of scattered, short, stout setae with rounded apices; basal half of inner margin with row of long, stout, hair-like setae; distal half almost without setae. Transverse row on the dorsal surface made of about 30 long, pointed apically stout setae (Fig. 11E, F). Dorsal surface of fore femur covered with scattered, short, stout setae and thin, hair-like setae. Dorsal surface of fore tibia with solitary hair-like setae and oblique regular row of long, stout, hair-like setae; outer margins of tibia with regular row of long, stout, hair-like setae.

Middle and hind femora, in contrast to fore femur, more slender, ca 1.8–2 times longer than wide, with denser submarginal row of short, stout setae (Fig. 11B, C). Outer and inner margins covered with a regular row of long, stout, hair-like setae. Outer margin of middle and hind tibiae with a regular row of long, stout, hair-like setae. Setation of dorsal surface of middle and hind tibiae similar to those of fore leg.

Tarsal claw moderately hooked, bearing 3–6 medial denticles, 1–2 subapical denticles (if two subapical denticles present, they are situated each on opposite sides of claw) and a row of 3–4 subapical setae on dorsal and ventral sides (Fig. 9G).

***Abdomen*.** All terga with median tubercles that bear short, stout setae with slightly divergent margins. Median tubercles moderately developed on terga IV–VIII, and slightly marked on terga I–III, IX, and X (Fig. 11H). Posterolateral projections moderately developed on segments VI–IX, and slightly marked on segments II–V. Submedian and sublateral areas of terga VI and VII with scattered stout setae with divided apices and a few small, rounded stout setae.

Gills on segments II–V (Fig. 9H–K); gill II with dorsal lamella operculate, oval and with entire margin; gills III–V with dorsal lamella incised medially.

Cerci length subequal to the body length; posterior margin of proximal half segments with elongate, stout setae with rounded apices; posterior margin of distal half segments with long, spine-like setae on the lateral margins; length of the stout setae less than length of corresponding segment. Paracercus absent.

***Egg.*** Ovoid, ca 100–110 μm long, with numerous micropyles. Egg with one polar cap, on the opposite pole with a cluster of 18–20 spines (Fig. 11I–K).

***Winged stages*.** Unknown.

##### Distribution.

Western Ghats (Tamil Nadu, India).

##### Habitats.

The larvae of *D.molinerii* sp. nov. inhabit cobble and pebble substrates of rivers with a strong current (Fig. 16E), where there is no significant anthropogenic stress. Water temperatures range between 20 and 22 °C and pH ranges between 7.1 and 7.4. This species was caught with other mayflies such as *Clypeocaenismalzacheri* Srinivasan, Sivaruban, Barathy & Isack, 2022 (Caenidae), *Nigrobaetisklugei* Sivaruban, Srinivasan, Barathy & Isack, 2022 (Baetidae), *Notophlebia* sp. (Leptophlebiidae), and *Tenuibaetisfrequentus* (Müller-Liebenau & Hubbard, 1985) (Baetidae).

##### Diagnosis.

*Dudgeodesmolinerii* sp. nov. can be distinguished from other *Dudgeodes* species by the following combination of characters. *Larva*: (i) dorsal part of male eyes dark brown to black; (ii) head with pair of small occipital tubercles; (iii) antennae length 1.25 times head width, flagellum with 11 segments; (iv) labrum with transversal row of scattered, stout, hair-like setae; (v) forefemur with transverse row of about 30 long, apically pointed, stout setae; (vi) tarsal claw bearing 3–6 medial denticles, and 1–2 subapical denticles (if two, they are on opposite sides of claw), and 3–4 subapical setae on dorsal and ventral sides; (vii) pronotum bears three pairs of tubercles: SMs, SLs, and Ls; mesonotum bears three pairs of tubercles: two pairs of SMMs, a pair of LAs, and unpaired MP; (viii) median tubercles moderately developed on terga IV–VIII, and slightly marked on terga I–III, IX and X; (ix) posterolateral projections moderately developed on segments VI–IX, and slightly marked on segments II–V. *Egg*: (i) egg with cluster of 18–20 spines present on pole opposite to polar cap; (ii) surface without microgranules.

Larval stage of this new species can be easily distinguished from other Indian *Dudgeodes* by: (i) presence of tubercles on head; (ii) number of tubercles on pro- and mesonotum; (iii) shape of femora; (iv) setation of forefemur; (v) size and shape median tubercles on abdomen.

#### ﻿ Teloganodes barathyae

Taxon classificationAnimaliaEphemeropteraTeloganodidae

Sivaruban, Martynov, Srinivasan & Isack sp. nov.

97ED1335-E7DD-5F11-975B-84FB65584920

https://zoobank.org/D430C0EB-5606-4AD4-9320-DBB8C546FF70

**Figure 12.**
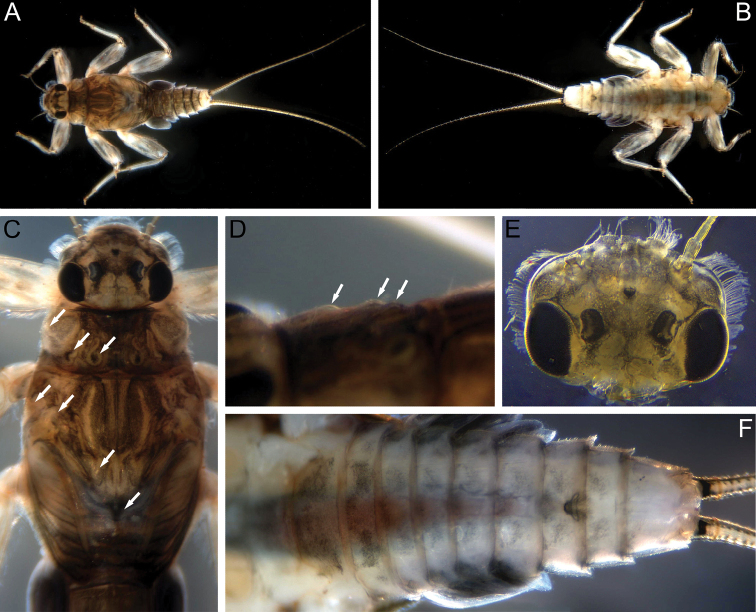
Larva of *Teloganodesbarathyae* Sivaruban, Martynov, Srinivasan & Isack, sp. nov., paratypes **A** total view, dorsal view **B** total view, ventral view **C** head and thorax, dorsal view **D** pronotum, dorso-lateral view **E** head, dorsal view **F** abdomen, ventral view. Abbreviations: white arrows show tubercles.

**Figure 13.**
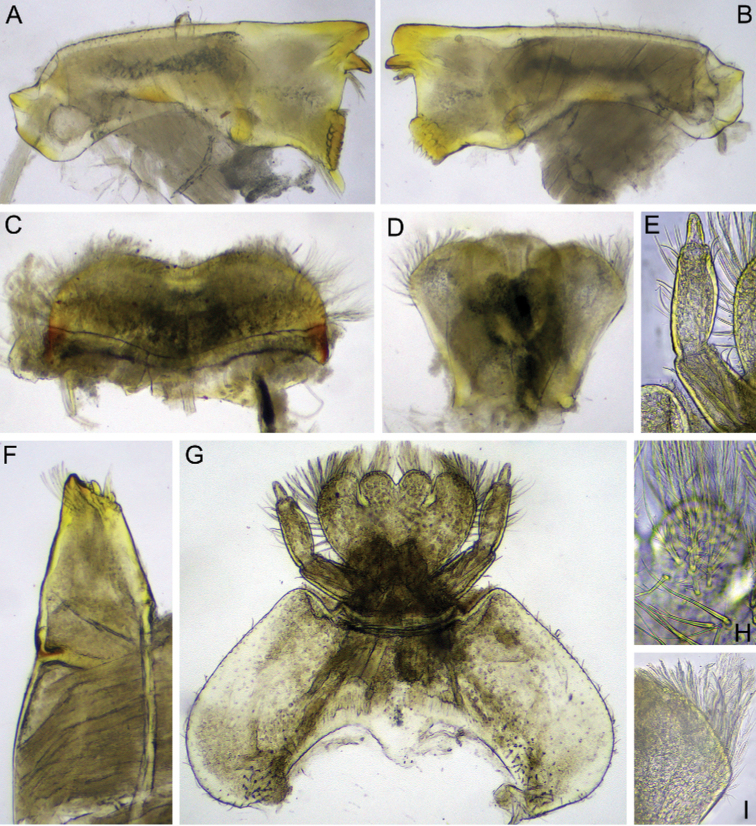
Larva of *Teloganodesbarathyae* Sivaruban, Martynov, Srinivasan & Isack, sp. nov., paratypes. **A, B** mandibles **C** labrum **D** hypopharynx **E** labial palp **F** maxilla **G** labium **H** stout setae on glossa **I** superlingua.

**Figure 14.**
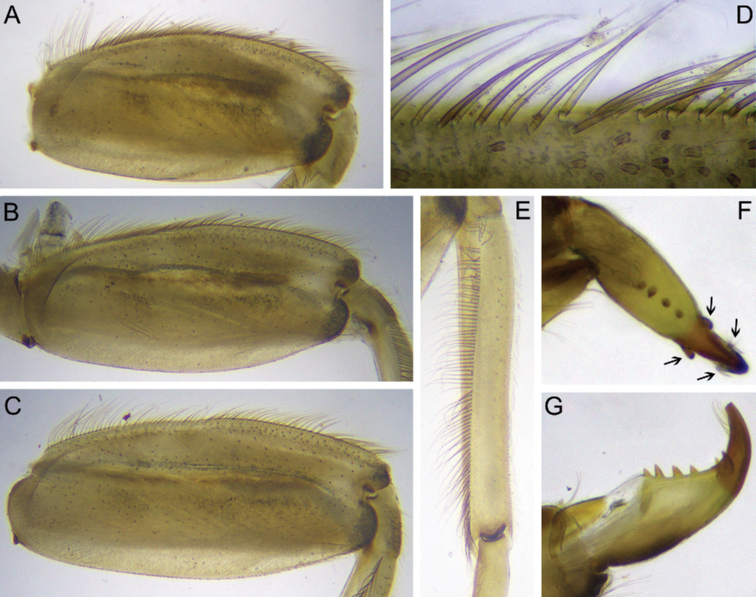
Larva of *Teloganodesbarathyae* Sivaruban, Martynov, Srinivasan & Isack, sp. nov., paratypes **A** fore femur **B** middle femur **C** hind femur **D** outer margin of hind femur **E** fore tibia **F, G** tarsal claw, ventral **F** and lateral **G** view. Abbreviations: black arrows show subapical denticles and setae.

**Figure 15.**
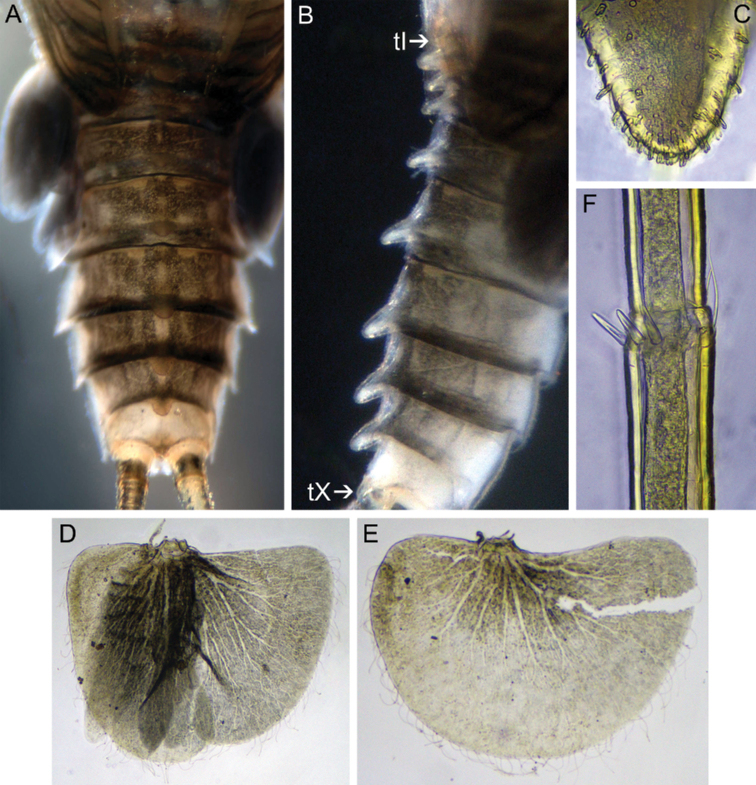
Larva of *Teloganodesbarathyae* Sivaruban, Martynov, Srinivasan & Isack, sp. nov., paratypes **A** abdomen, dorsal view **B** abdomen, lateral view **C** median tubercle of abdominal tergum VI **D** gill V **E** gill VI **F** setae of caudal filament.

Figs 12 , 13 , 14 , 15

##### Material examined.

***Holotype***: ♀ larva, India, Tamil Nadu, Theni District, Kurangani Hills, Kottakudi River, 10.0809°N, 77.2552°E, 632 m a.s.l., 28.x.2020, Pandiarajan Srinivasan & Isack Rajasekaran leg., ZSI–SRC/I/E/652. ***Paratypes***: 2 larvae, ibid., 28.x.2020, Pandiarajan Srinivasan & Isack Rajasekaran leg., ZSI–SRC/I/E/653 (1 larva), AMC ZN 230 (1 larva).

##### Etymology.

The new species is named in honour of Dr S. Barathy, an assistant professor in the Department of Zoology, Fatima College, Tamil Nadu, India, who contributed to the study aquatic insects of India.

##### Description.

***Mature larva*.** Body length up to 5.4–5.7 mm without cerci; cerci length subequal to body length. General colouration of dorsal side of head, thorax, and terga I–IX brownish to blackish; tergum X yellowish to whitish; ventral side of the head and thorax pale, sterna I–VIII with submedian black tinges; sternum IX pale (Fig. 12A–C, F); legs light brownish; femora light brownish with two distinct maculae (Fig. 14A–C); basal segment of cerci black; apical parts of cerci somewhat blacked out.

***Head*.** Lateral margins of head fringed with a row of long, stout setae, forked near base and with pointed apices, which run from posterior margin of eyes to labrum; anterior margin of clypeus with numerous stout setae of the same type (Fig. 12E). Antennae short, 0.8 times head width, flagellum with 13–14 segments. Dorsal part of male eyes reddish.

***Mouthparts*.** Labrum compact, ca 2.4 times wider than long, with smooth anterior emargination; dorsal surface with a transversal band of numerous feathered setae (Fig. 13C). Mandibles slender; middle of outer margin with one long, stout seta or without seta (when absent, probably broken). Right mandible (Fig. 13A) with inner incisor composed of two teeth; prostheca reduced, comprised of a cluster of thin setae; a small row of six long, stout, hair-like setae below mola and a bunch of short, thin setae above mola. Left mandible (Fig. 13B) inner incisor with two teeth inserted transversely, one smaller and pointed, other large and rectangular; prostheca small with three short and long setae; no setae below mola. Number of teeth of both mandibular outer incisors undetermined due to their wear in type specimens. Maxilla (Fig. 13F) slender, canine completely worn, its shape undetermined, two dentisetae serrated on the inner margin and three long setae on inner apical region, and cluster of long, simple setae at crown; inner margin at the base of lacinia, with a long, feathered seta dorsally and 4–5 setae of the same type ventrally; maxillary palp greatly reduced up to protuberance with seta. Superlinguae laterally angular, with a row of long, feathered setae at apex (Fig. 13D, I). Submentum well developed laterally; glossae and paraglossae partially fused; paraglossae larger than glossae; labial palp three-segmented, articulation between all three segments well visible; segments I and II subequal in length; segment III ca 1.8 times as long as wide (Fig. 13E, G, H).

***Thorax*.** Pronotum with three pairs of rounded tubercles: SMs, SLs, and Ls. Mesonotum with three pairs of tubercles: two pairs of SMMs and LAs (lateral anterior tubercles), and an unpaired MP tubercle (Fig. 12C, D).

Forefemur (Fig. 14A) moderately broad, ca 2.4 times longer than wide; outer margin with a regular row of long, stout, hair-like setae. Dorsal surface with submarginal row of numerous short, stout setae with divergent margins (some of setae divided near apex into two rounded lobes); same stout setae scattered over whole dorsal surface; inner margin with a regular row of long, stout hair-like setae, this row continuing on dorsal surface to near articulation with trochanter; transverse row of stout setae absent. Dorsal surface of fore tibia with oblique regular row of long, stout, hair-like setae and solitary hair-like setae; outer margins of tibia with a regular row of long, stout, hair-like setae (Fig. 14E).

Middle and hind femora with ornamentation similar to foreleg (Fig. 14B–D). Middle and hind tibiae with a row of long and stout, hair-like setae on outer margin; dorsal surface with oblique row of long, stout, hair-like setae; also scattered short, stout setae with divergent margins (some of the setae divided near apex into two rounded lobes) present on dorsal surface along outer margin.

Tarsal claw hooked, bearing four medial denticles and two subapical denticles on opposite sides of claw; dorsal and ventral surface of claw with a row of 3–5 subapical, hair-like setae (Fig. 14F, G).

***Abdomen*.** Median tubercles on terga I–X present; on tergum I poorly developed; on terga II–IV moderately developed; on terga V–X most developed. In dorsal view tubercles I–IX broad and rounded apically, tubercle X distinctly slender and bluntly pointed (Fig. 15A, B). Median tubercles with short, stout setae (Fig. 15C). Posterolateral projections present on segments II–IX; slightly marked on segments I–VI; most distinct on segments VII–IX (Fig. 12F). Submedian and sublateral areas of terga VI and VII with differently sized (mainly medium-length and long) stout setae with slightly or moderately divided apices.

Gills present on abdominal segments II–VI. Gill II with dorsal lamella operculate and covering others, oval with margin entire; gills II–V with well-developed flabelliform ventral lobe; gills III–V with dorsal lamella incised medially (Fig. 15D); gill VI with dorsal lamella entire (Fig. 15E).

Central portion of cerci with elongate stout setae with bluntly pointed apices and few long, hair-like setae; stout setae length less than half length of the corresponding segment (Fig. 15F).

***Winged stages*.** Unknown.

##### Distribution.

Western Ghats (Tamil Nadu, India).

##### Habitat.

The same as for *D.molinerii* sp. nov.

##### Diagnosis.

Larva of *T. barathyae* sp. nov. can be distinguished from other species of *Teloganodes* by the following combination of characters: (i) dorsal surface of labrum with a transversal band of numerous feathered setae; (ii) inner incisor of the left mandible with two teeth inserted transversely, one smaller and pointed, the other large and rectangular; (iii) superlinguae angular laterally, with a row of long, feathered setae at apex; (iv) forefemur moderately broad, ca 2.4 times longer than wide; outer margin with regular row of long, stout, hair-like setae; without any combination of thin and stout setae in a row; (v) forefemur bears submarginal row of numerous short stout setae with divergent margins, (some of them divided near apex into two rounded lobes); same stout setae scattered over whole dorsal surface; (vi) fore femur without transverse row of stout setae; (vii) median tubercles on terga I–X, on tergum I poorly developed; on terga II–IV moderately developed; on terga V–X best developed; in dorsal view tubercles I–IX broad and rounded apically, tubercle X distinctly slender and bluntly pointed; (viii) posterolateral projections on segments II–IX, segments VII–IX well developed but not extremely.

**Figure 16.**
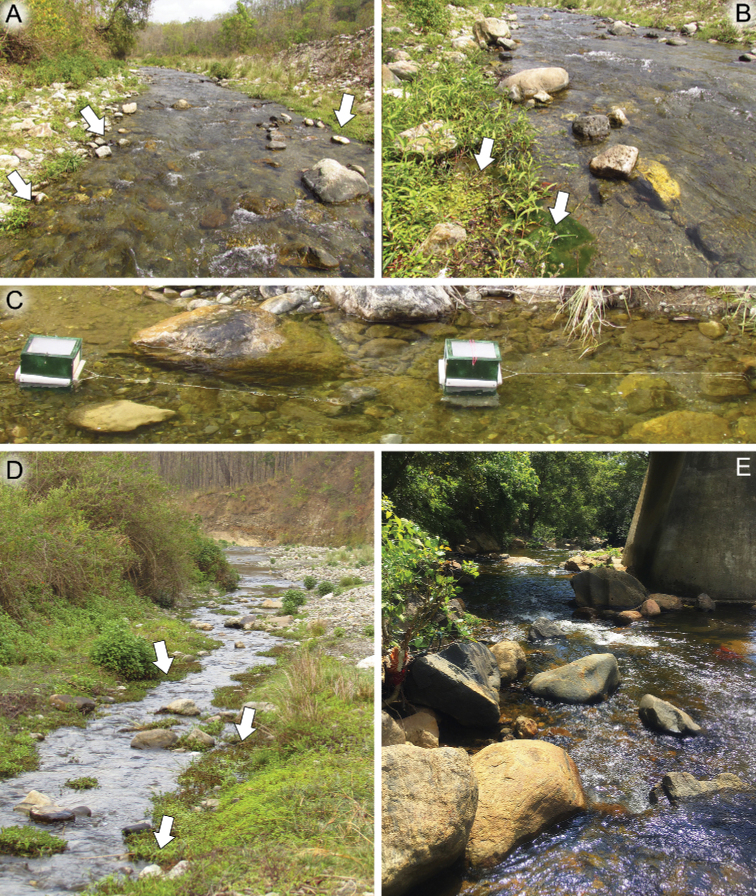
Type habitats **A, B, D, E** of new species and cages (Martynov’s construction) **C** used for *Dudgeodesselvakumari* Martynov & Palatov, sp. nov. winged stages rearing **A, B, D** type habitat of *Dudgeodesselvakumari* Martynov & Palatov, sp. nov. **C** Martynov-designed grow nets for mayfly winged stages rearing **E** type habitat of *Dudgeodesmolinerii* Sivaruban, Martynov, Srinivasan, Barathy & Isack, sp. nov. and *Teloganodesbarathyae* Sivaruban, Martynov, Srinivasan & Isack, sp. nov. Abbreviations: arrows show microhabitats with the highest density of a new species’ larvae.

Larvae of this new species can be distinguished for other Indian representatives of *Teloganodes* by: (i) shape of superlinguae; (ii) length of antennae; (iii) absence of transversal row of stout setae on forefemur; (iv) shape of forefemur; (v) shape of median tubercles of abdominal terga.

#### ﻿ Teloganodes

Taxon classificationAnimaliaEphemeropteraTeloganodidae

sp. IND1

7B0CCDA2-2EE1-509D-8E07-CCE45065C2B2

##### Remark.

This operational taxonomic unit is known by one specimen which distinctly differs from other representatives of the genus. We consider this material unacceptable for describing a new species now but provide a diagnosis.

##### Material examined.

1 larva, India, Tamil Nadu, Theni District, Kurangani Hills, Kottakudi River, 10.0809°N, 77.2552°E, 632 m a.s.l., 28.x.2020, Pandiarajan Srinivasan & Isack Rajasekaran leg., AMC ZN 243.

##### Diagnosis.

This OTU can be distinguished from other representatives of *Teloganodes* by the following combination of characters: (i) superlinguae laterally angular; (ii) forefemur without transversal row of stout setae; (iii) outer margin of femora with a regular row of long, stout hair-like setae only; (iv) dorsal surface of femora with submarginal irregular row of short stout setae; (v) tarsal claw with four median denticles and 1–2 subapical denticles (if two, on opposite sides of claw); (vi) median tubercles on terga III–X, which are indistinct on tergum III; wide on terga IV–VI; largest and elongate on terga VII–IX; elongate and thin on terga X; (vii) sublateral areas of posterior margins of terga IV–VI with bunches of 3–4 extremely long, pointed, stout setae; (viii) surface of cerci covered with long, stout, hair-like setae; setae in posterior margin of cerci segments less than half length of corresponding segment.

### ﻿Molecular results

In this study, we used all available COI sequences of *Dudgeodes* species. Notably, 10 *Dudgeodes* OTUs, which have not yet been described morphologically, have already been sequenced and their independent position is certain (Garces et al. 2020; GenBank data); these are included in our ML tree.

**Figure 17.**
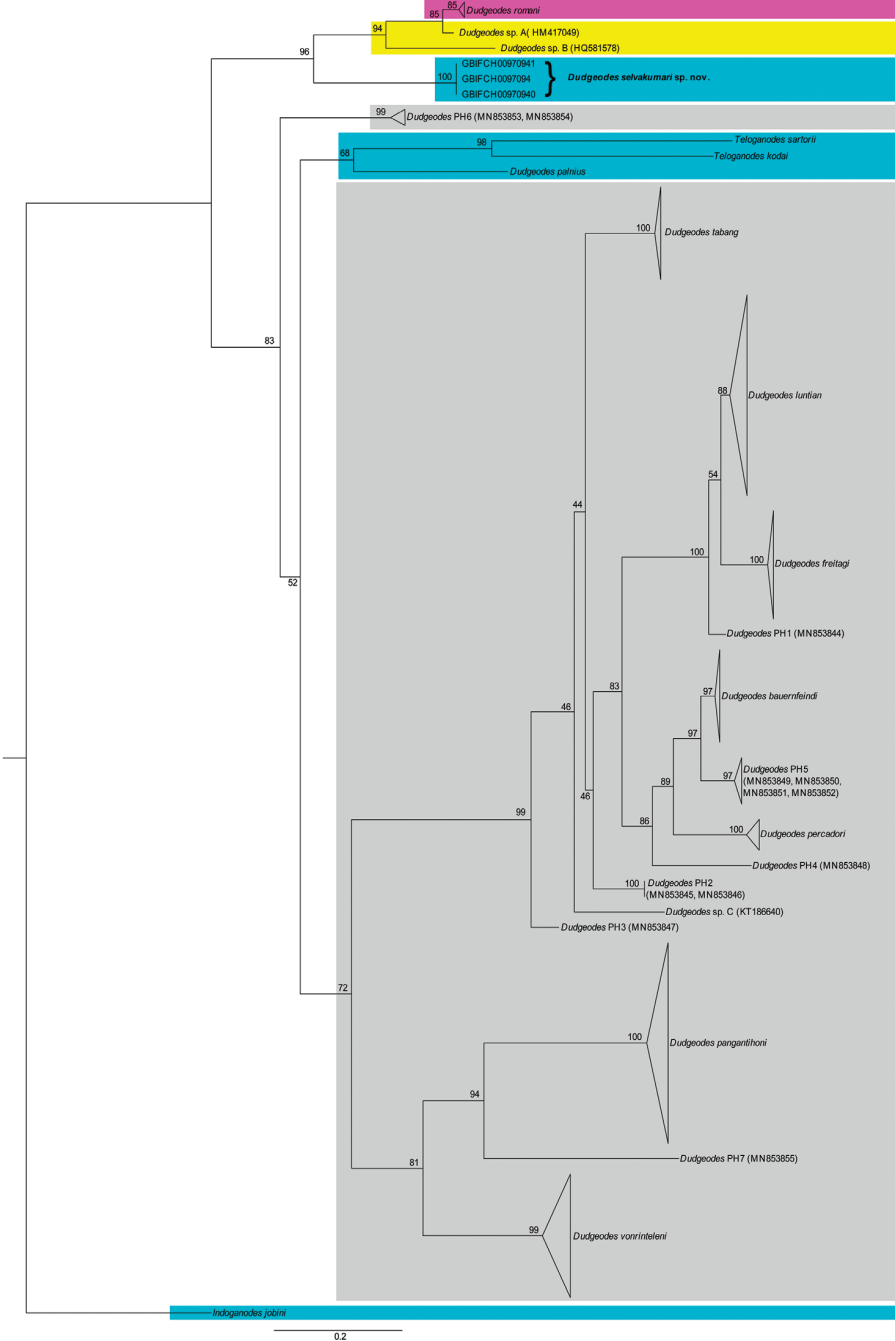
Maximum-likelihood tree including several representatives of the genus *Dudgeodes* and *Teloganodes*. Branches provided with bootstrap supports (BS). Abbreviations: pink –Cambodia, yellow – Thailand, blue – India, gray – Philippines.

All sequenced species of *Dudgeodes*, including undescribed OTUs, from continental Southeast Asia, excluding *D.palnius*, form a separate clade (Fig. 17). The phylogenetic reconstruction based of the COI gene supports *D.selvakumari* sp. nov. as a monophyletic clade with a bootstrap support of 100%. Genetic distances within the species is 0.00 (*n* = 3), which is partially due to all specimens having come from a single locality. Genetic distances between the new species and three other most related species according to ML tree (*D.romani*, *D.* sp. A, and *D.* sp. B) are large (0.43–0.46; Table 2). Notably that genetic distance, calculated using the Tamura-Nei with a gamma distribution, between *Dudgeodespalnius* and the clade of *D.selvakumari* sp. nov., *D.romani*, *D.* sp. A, and *D.* sp. B, that is not closely related to *D.palnius* according to the ML tree (genetic distance 0.70–0.84). There are also high genetic distances with these taxa using the Kimura 2-parameter model with a gamma distribution. These results cannot be explained and, in our opinion, mistakes in *D.palnius* sequencing cannot be excluded.

**Table 2.** Genetic distances (COI) between sequenced *Dudgeodes* species of continental part of Southeast Asia, calculated using the Tamura-Nei (TN93) and Kimura 2-parameter (K2) models with a gamma distribution (G) (TN93+G/K2+G).

	*Dudgeodesromani*	*Dudgeodes* sp. B (HQ581578)	*Dudgeodes* sp. A (HM417049)	*Dudgeodesselvakumari* sp. nov.
*Dudgeodesromani*	0.01/0.01			
*Dudgeodes* sp. B (HQ581578)	0.3226/0.3695	—		
*Dudgeodes* sp. A (HM417049)	0.0558/0.0545	0.3037/0.3585	—	
*Dudgeodesselvakumari* sp. nov.	0.4616/0.5456	0.4642/0.5376	0.4310/0.5140	0.00/0.00

## Supplementary Material

XML Treatment for Dudgeodes selvakumari

XML Treatment for Dudgeodes molinerii

XML Treatment for Teloganodes barathyae

XML Treatment for Teloganodes
